# Additive Effects of Quorum Sensing Anti-Activators on *Pseudomonas aeruginosa* Virulence Traits and Transcriptome

**DOI:** 10.3389/fmicb.2017.02654

**Published:** 2018-01-09

**Authors:** Kyle L. Asfahl, Martin Schuster

**Affiliations:** Department of Microbiology, Oregon State University, Corvallis, OR, United States

**Keywords:** quorum sensing, acyl-homoserine lactone, gene expression, RNA-seq/transcriptomics, anti-activator, *Pseudomonas aeruginosa*

## Abstract

In the opportunistic pathogen *Pseudomonas aeruginosa*, quorum sensing (QS) via acyl-homoserine lactone (AHL) signals coordinates virulence gene expression. AHL signals must reach a critical threshold before enough is bound by cognate regulators LasR and RhlR to drive transcription of target genes. In addition, three anti-activator proteins, QteE, QscR, and QslA, sequester QS regulators to increase the threshold for induction and delay expression of QS target genes. It remains unclear how multiple anti-activators work together to achieve the quorum threshold. Here, we employed a combination of mutational, kinetic, phenotypic, and transcriptomic analysis to examine regulatory effects and interactions of the three distinct anti-activators. We observed combinatorial, additive effects on QS gene expression. As measured by reporter gene fusion, individual deletion of each anti-activator gene increased *lasB* expression and QS-controlled virulence factor production. Deletion of *qslA* in combination with the deletion of any other anti-activator gene resulted in the greatest increase and earliest activation of *lasB* gene expression. Western analysis revealed that relative increases in soluble LasR in anti-activator mutants correlate with increased *lasB* expression and QS-controlled virulence factor production. RNA-seq of the previously uncharacterized QslA and QteE regulons revealed overlapping, yet distinct groups of differentially expressed genes. Simultaneous inactivation of *qteE* and *qslA* had the largest effect on gene expression with 999 genes induced and 798 genes repressed in the double mutant vs. wild-type. We found that LasR and RhlR-activated QS genes formed a subset of the genes induced in the *qteE, qslA*, and double mutant. The activation of almost all of these QS genes was advanced from stationary phase to log phase in the *qteE qslA* double mutant. Taken together, our results identify additive effects of anti-activation on QS gene expression, likely via LasR and RhlR, but do not rule out QS-independent effects.

## Introduction

Bacterial cell-cell signaling is a widespread mechanism of communication, allowing coordination of behavior among cells in a population (Waters and Bassler, 2005). This intercellular signaling is generally termed quorum sensing (QS), but the signaling mechanisms and behaviors regulated by QS in different bacteria are diverse (Cook and Federle, 2014; Asfahl and Schuster, 2017). The environmental bacterium and opportunistic human pathogen *Pseudomonas aeruginosa* has been established as a premier model system for studying QS regulation via diffusible acyl-homoserine lactone (AHL) signals. Hundreds of target genes, many encoding secreted virulence factors, are controlled through a hierarchy of two complete, interconnected LuxI/R-type AHL circuits (Hentzer et al., 2003; Schuster et al., 2003; Wagner et al., 2003). The LasI synthase produces the *N*-3-oxo-dodecanoyl-homoserine lactone (3OC12-HSL) signal bound by the LasR receptor-regulator (an “R-protein”), and the RhlI synthase produces the *N*-butanoyl-homoserine lactone (C4-HSL) signal bound by the RhlR receptor-regulator (Schuster and Greenberg, 2006). 3OC12-HSL-dependent homodimerization of LasR produces an active regulatory complex capable of binding DNA and promoting transcription (Kiratisin et al., 2002). While RhlR may form homodimers in the absence of the C4-HSL signal, RhlR-dimer binding of signal is required for DNA binding and transcriptional activity (Ventre et al., 2003). Signal-bound LasR and RhlR function as transcriptional activators by binding to conserved sequence elements upstream of target promoters. LasR also activates the expression of *rhlR*, linking the two QS systems (Latifi et al., 1996; Pesci et al., 1997).

Most QS target genes are activated at the beginning of stationary phase in batch culture (Schuster et al., 2003). QS signal accumulation is necessary but not sufficient for QS gene induction. Additional regulatory inputs, such as the general stress response, are required for expression and in part shape the content of the QS regulon at high cell densities (Schuster et al., 2004a; Schuster and Greenberg, 2006; Williams and Camara, 2009). Opposing QS activation in the presence of signal, anti-activation of QS components can effectively delay the QS response (Asfahl and Schuster, 2017). Anti-activation was originally discovered in *Agrobacterium tumefaciens*, where a TraM anti-activator protein binds to and sequesters the LuxR-type receptor TraR, suppressing AHL-QS activation and transcription of TraR target genes (Fuqua et al., 1995; Hwang et al., 1995). Deletion of *A. tumefaciens* TraM activates QS at a much lower cell density (Hwang et al., 1995), possibly representing constitutive activation. It has therefore been proposed that anti-activation could prevent intracellular self-activation of receptors, also termed “short-circuiting” (Goryachev et al., 2005). In this model, the stoichiometry of LuxR-type receptors with anti-activators determines the induction threshold. More generally, anti-activation may tune the induction threshold to optimize the benefits attained from costly secretions (Pai et al., 2012; Gupta and Schuster, 2013).

**Figure 1:**
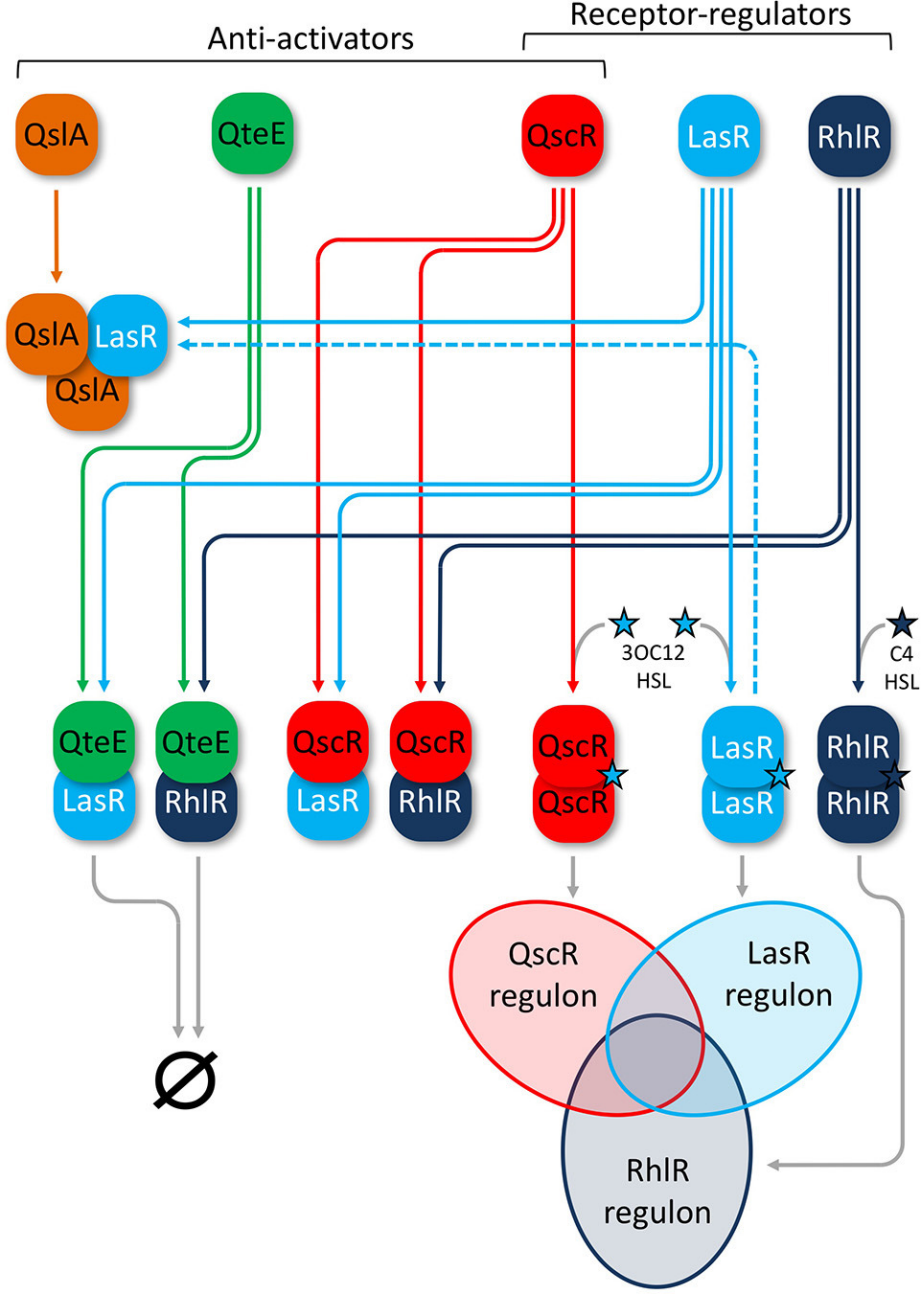
Anti-activators in the *P. aeruginosa* AHL-QS circuitry. QS receptor-regulators (white labels) LasR (light blue) and RhlR (dark blue) homodimerize upon cognate signal binding of 3OC12-HSL (Las, light blue star) or C4-HSL (Rhl, dark blue star), leading to activation of overlapping LasR and RhlR regulons, respectively (Venn diagram). Anti-activators (black labels) QscR (red), QteE (green), and QslA (orange) all interact with LasR and RhlR to suppress QS activation. QteE likely forms heteromultimers with both LasR and RhlR separately, leading to degradation of each regulator. The exact stoichiometry of QteE-R-protein complexes is not known. QslA is only known to form heterotrimers with LasR in a 2:1 ratio, but the overall fate of this complex beyond suppression of QS is not entirely clear. QslA can also dissociate and then bind previously formed LasR homodimers (dashed line). QscR appears to form heterodimers with both LasR and RhlR, but also acts as an orphan receptor-regulator that can homodimerize upon binding of 3OC12-HSL and regulate its own overlapping QS regulon (Venn diagram). Venn diagram lobes are not scaled to reflect relative size of each regulon.

Three anti-activator proteins that work to suppress QS-activation through R-proteins have been identified in *P. aeruginosa* thus far: QteE, QscR, and QslA (Figure 1). The orphan LuxR homolog QscR (PA1898) has been observed in the formation of heteromultimeric complexes with both LasR and RhlR (Ledgham et al., 2003). QscR suppresses quorum-controlled operons involved in hydrogen cyanide and phenazine biosynthesis (Chugani et al., 2001). Microarray analysis showed that *qscR* represses 329 genes, although it also induces a small, separate set of target genes (Lequette et al., 2006). Induction of at least some genes is through the additional function of QscR as a 3OC12-HSL-responsive transcriptional activator that dimerizes upon signal binding (Lee et al., 2006; Lequette et al., 2006; Lintz et al., 2011). The structurally unrelated anti-activator protein QteE (PA2593) may also form a heterodimer with LasR that prevents signal binding and destabilizes LasR (Siehnel et al., 2010). The authors of that study also found that in addition to LasR, QteE can reduce RhlR QS-transcriptional activity independently, as well as destabilize the RhlR protein (Siehnel et al., 2010). A third protein, QslA (PA1244), acts as a potent anti-activator of LasR through heterotrimer formation that can even dissociate previously formed LasR-DNA complexes (Seet and Zhang, 2011). This effect is achieved through direct binding of QslA to the ligand-binding-domain (LBD) of LasR in a 2:1 ratio, obscuring the dimerization interface and thereby preventing activation (Fan et al., 2013).

Despite the contributions of these studies, the roles of individual anti-activators and the scope of their collective influence on the QS induction threshold are not fully understood. Gupta and Schuster found that mutations in either *qteE* or *qscR* can produce virtually identical phenotypes under certain conditions (Gupta and Schuster, 2013). QteE, QscR, and QslA are not homologous and may bind R-proteins differently (Siehnel et al., 2010; Lintz et al., 2011; Fan et al., 2013). In consideration of this evidence together, several important open questions remain. Why would *P. aeruginosa* maintain multiple, similarly functioning anti-activators? Does deletion of multiple anti-activators produce a stronger affect than loss of a single gene, and how do different anti-activators affect the QS regulon? Deletion of any single anti-activator is sufficient to produce a general increase in QS gene expression (Chugani et al., 2001; Siehnel et al., 2010; Seet and Zhang, 2011; Gupta and Schuster, 2013), indicating their functions are not completely redundant. Interactions between anti-activators are possible, a scenario that could produce additive or synergistic effects. Given current mechanistic information, it is plausible that most, if not all of the genes affected by anti-activator deletions are those activated by LasR and RhlR. Here we use mutational analysis of anti-activator genes in combination with phenotypic measurements, gene induction kinetics, and transcriptome profiling to address these questions.

## Materials and methods

### Strains and culture conditions

See Table 1 for a comprehensive list of strains and plasmids. *Pseudomonas aeruginosa* PAO1 was used as the wild-type isogenic parent in mutant construction and as the control strain in all experiments. PAO1 and the isogenic, markerless Δ*qteE* and Δ*lasR* Δ*rhlR* (DA6) knockouts were obtained from R. Siehnel (Univ. Washington, USA) (Siehnel et al., 2010). The Δ*qslA* and Δ*qslA* Δ*qteE* mutants were created using a pEX18-based suicide vector (Hoang et al., 1998). We subcloned an in-frame deletion constructed by splicing-overlap-extension PCR (SOE-PCR) into pEX18Gm for use in allelic exchange (Horton, 1995; Hoang et al., 1998). The PAO-R3 (*qscR*-Gm^R^) strain was obtained from S. Chugani (Univ. Washington, USA) (Chugani et al., 2001). This strain, as well as the strains obtained from R. Siehnel, are based on the “Iglewski” lineage of PAO1. The PAO Δ*qteE qscR*-Gm^R^ and PAO Δ*qteE* Δ*qslA qscR*-Gm^R^ strains were created by introducing genomic DNA from strain PAO-R3 harboring the *qscR*-Gm^R^ allele into PAO Δ*qteE* and PAO Δ*qteE* Δ*qslA* via whole-genome transformation (Choi et al., 2006).

**Table 1:** Bacterial strains and plasmids.

**Strain or plasmid**	**Relevant properties**	**References or origin**
***Pseudomonas aeruginosa***		
PAO1	Wild-type, PAO1 UW library strain (originally from B. Iglewski, Rochester)	Jacobs et al., 2003
PAO Δ*qteE*	PAO1 derivative; markerless *qteE* deletion mutant; “*qteE*”	Siehnel et al., 2010
PAOR3	PAO1 derivative; *qscR*-Gm^R^, null mutant marked with Gm cassette inactivating *qscR*; “*qscR*”	Chugani et al., 2001
PAO Δ*qslA*	PAO1 derivative; Δ*qslA*, unmarked in-frame deletion from amino acid 6 to 111; “*qslA*”	This study
PAO Δ*qteE* Δ*qslA*	PAO1 Δ*qteE* derivative; unmarked double-null deletion mutant in which both *qteE* and *qslA* harbor in-frame deletions; “*qteE qslA*”	This study
PAO *qscR*-Gm^R^ Δ*qslA*	PAOR3 derivative; marked double-null mutant which harbors both Δ*qslA* and *qscR*-Gm^R^ alleles; “*qscR qslA*”	This study
PAO Δ*qteE qscR*-Gm^R^	PAO1 Δ*qteE* derivative; marked double-null mutant harbors both Δ*qteE* and *qscR*-Gm^R^ alleles; “*qteE qscR*”	This study
PAO Δ*qteE* Δ*qslA qscR*-Gm^R^	PAO1 Δ*qteE* Δ*qslA* derivative; marked triple-null mutant harbors Δ*qteE*, Δ*qslA*, and *qscR*-Gm^R^ alleles; “*qteE qslA qscR*”	This study
DA6	PAO1 derivative; Δ*lasR* Δ*rhlR*,unmarked double-null deletion mutant in which both *lasR* and *rhlR* harbor in-frame deletions; “*lasR rhlR*”	Siehnel et al., 2010
***Escherichia coli***		
DH5α	F^−^Φ80*lacZYA-argF* U169 *recA1 hsdR17* (${\text{r}}_{\text{k}}^{-}$, ${\text{m}}_{\text{k}}^{+}$) *phoA supE44* λ^−^*thi-1 gyrA96 relA1*	Invitrogen
SM10	*thi thr leu tonA lacY supE recA*::RP4-2-Tc::Mu Km^R^ λ*pir*	Simon et al., 1983
***Plasmids***		
pEX18Gm	Conjugative suicide plasmid; Gm^R^	Hoang et al., 1998
pEX18Gm.Δ*qslA*	pEX18Gm with Δ*qslA* containing an in-frame deletion from amino acid 6 to 111	This study
pProbeAT	Broad-host-range vector with a promoterless *gfp*, Cb^R^	Miller et al., 2000
pRG13	240 bp *lasB* promoter cloned into pProbeAT	Gupta and Schuster, 2013

All routine and experimental cultures were grown at 37°C. For routine propagation, we grew strains stationary on Lennox LB agar or with shaking at 250 rpm in Lennox LB broth buffered with 50 mM 3-(*N*-morpholino)-propanesulfonic acid (MOPS), pH 7.0. When necessary, plates were supplemented with 100 μg/ml tetracycline or 100 μg/ml gentamicin for the selection of marked strains. Strains containing reporter plasmids were grown with 200 μg/ml carbenicillin in routine cultures, but not in experimental cultures. When necessary, cells were washed, resuspended, and diluted in M9 minimal medium with no carbon added (M9-salts) (Gupta and Schuster, 2013). For inoculation of all experimental cultures, we modified a previously described recursive growth-dilution pre-culture scheme (Siehnel et al., 2010; Gupta and Schuster, 2013) to effectively dilute carryover GFP-fluorescence from previously induced *P*_*lasB*_-*gfp* reporter gene expression. First, fresh colonies from plates were suspended in M9-salts, optical density was measured at 600 nm (OD_600_, reported as 1 cm pathlength) and then diluted to allow initial inoculation of 4 ml LB-MOPS at OD_600_ = 0.0001 in glass culture tubes. After incubation at 37°C with shaking, cells were harvested in exponential (log) phase (OD_600_ < 0.2), washed in M9-salts, and re-diluted into 4 ml fresh LB-MOPS at OD_600_ = 0.0000001. After another incubation at 37°C with shaking, cells were again harvested in log phase (OD_600_ < 0.2), washed in M9-salts, and then diluted to compose experimental inocula. For transcriptional reporter assays, endpoint phenotypic assays, and transcriptomic analysis, M9 minimal medium was supplemented with 0.5% (w/v) casamino acids (CAA) as the sole carbon source (Gupta and Schuster, 2013). CAA medium serves as a semi-defined medium in which all required amino acids are present, reflecting the products of proteolytic activity, and as such representing nutritional conditions likely encountered by *P. aeruginosa* in the environment. In addition, this medium exhibits significantly lower autofluorescence compared with LB, allowing precise quantitation of GFP fluorescence from transcriptional reporters. Growth experiments conducted in the plate reader were terminated at 800 min due to evaporation in this configuration and corresponding increased variability beyond this time point. All experiments were performed using a minimum of three biological replicates with independently prepared inocula.

### GFP-transcriptional reporter assays

A plasmid-borne fusion of the QS-controlled *lasB* (PA3724) promoter sequence (240 bp) and GFP was used to assess promoter activity in our collection of mutants. We used fluorescence spectroscopy for detection as previously described (Gupta and Schuster, 2013). Briefly, pRG13 (*P*_*lasB*_-*gfp*) and pProbeAT (promoterless *gfp* negative control) were individually introduced into each strain background. Following our recursive growth-dilution scheme, precultured cells were inoculated at a starting OD_600_ = 0.01 in 200 μL of CAA medium in black-walled 96-well plates with a clear bottom (Greiner bio-one, Cat. No. 655090). Cell density (absorbance at 600 nm, reported as OD_600_, 1 cm pathlength) and fluorescence (GFP, λ_excitation_ = 480 nm, λ_emission_ = 535 nm, gain setting = 60) were measured in 15 min intervals as cultures were incubated with shaking at 37°C in a Tecan Infinite M200 multifunction plate reader. *P*_*lasB*_-*gfp* promoter activity for individual strains was corrected for background fluorescence by subtracting the OD-normalized fluorescence of a strain harboring pProbeAT from the OD-normalized fluorescence of the corresponding strains with the active reporter for each time point. *P*_*lasB*_-*gfp* expression rates were calculated as the time derivative of OD-normalized GFP fluorescence [d(GFP/OD)/dt] over a 30 min period as described previously (Gupta and Schuster, 2013). Data were smoothed by reporting the mean of three consecutive measurements.

### Pyocyanin production assay

Pyocyanin production of individual strains was assessed essentially as described previously (Essar et al., 1990; Mellbye and Schuster, 2014). Starting with our recursive dilution scheme, we inoculated each precultured strain into 5 ml CAA medium at a starting OD_600_ = 0.01 and allowed cultures to grow with shaking at 37°C for 18 h (stationary phase, OD_600_ = 1.9–2.3). Pyocyanin was extracted from 5 ml supernatant using 3 ml chloroform, followed by addition of 1 ml 0.2 M HCl to the chloroform phase. After separation of the acidified pyocyanin from the top of the mixture, A_520_ of 200 μl aliquots was measured in a Tecan plate reader and reported as fold-change vs. wild-type production.

### Elastase activity assay

Elastolytic activity of stationary phase supernatants was determined using the elastin congo red (ECR) assay as previously described (Diggle et al., 2002), but modified to allow high throughput. Starting with our pre-culture scheme, we inoculated each strain into 800 μl CAA medium at a starting OD_600_ = 0.01 and allowed cultures to grow at 37°C with shaking in 96-well deep-well blocks (VWR North America, Cat. No. 82006-448) covered with Breathe Easy® sealing membranes (Diversified Biotech, Cat. No. BEM-1). After 18 h, OD_600_ was measured in a Tecan plate reader (stationary phase, OD_600_ = 1.9–2.3). Cells were pelleted at 4,000 rpm for 10 min, followed by sterile filtration of 250 μl supernatant in AcroPrep™ 96-well filter plates (Pall Life Sciences, Cat. No. 5045). Forty μl cell-free supernatant was combined with 360 μl ECR buffer (100 mM Tris, 1 mM CaCl_2_, pH 7.5) containing 20 mg/ml ECR (Sigma-Aldrich Co., Cat. No. E0502) in sealed 96-well deep-well blocks and incubated at 37°C with shaking for 3 h. After pelleting insoluble ECR at 4,000 rpm for 10 min, 200 μl supernatant was transferred to a 96-well plate for measurement of absorbance at 495 nm in a Tecan plate reader. Elastolytic activity of supernatants is reported as fold-change vs. wild-type activity.

### LasR western analysis

Relative LasR concentrations were determined following an established Western blot protocol (Schuster and Greenberg, 2007). Starting with our recursive dilution scheme, we inoculated each strain into 4 ml CAA medium at a starting OD_600_ = 0.01 and incubated cultures with shaking at 37°C, periodically measuring OD_600_ to monitor growth. Cells were pelleted at OD_600_ values of 0.2 (log phase) and 1.6 (early stationary phase), and pellets were frozen at −80°C until processing. Pellets were resuspended in lysis buffer without added signals, followed by sonication. Insoluble debris was pelleted by centrifugation at 13,000 × g. Soluble protein concentrations of lysates were determined using the Bio-Rad Protein Assay (Bradford; Bio-Rad, Cat. No. 5000205) in microplate format. Approximately 5 μg total protein from each sample was separated by 12.5% sodium dodecyl sulfate-polyacrylamide gel electrophoresis. Separated proteins were blotted onto nitrocellulose membranes (Bio-Rad, Cat. No. 1620148), probed with polyclonal anti-LasR antibodies (Schuster and Greenberg, 2007), and then visualized using chemiluminescence detection reagents (GE Healthcare, Cat. No. RPN2209) and autoradiography film. Densitometric analysis of exposed films was carried out using the gel analysis function in ImageJ (https://imagej.nih.gov/ij/).

### RNA sequencing transcriptome generation

RNA sequencing (RNA-seq) was carried out on a subset of five of our strains; WT, *lasR rhlR, qteE, qslA*, and *qteE qslA* were each examined at two time-points with three biological replicates made from separate preparations on separate days, producing a total of 30 samples. Cultures were prepared and grown exactly as described in the previous section on LasR Western Analysis, above. Approximately 2 × 10^9^ cells were harvested at OD_600_ values of 0.2 (log phase) and 1.6 (early stationary phase), immediately preserved using RNAprotect Bacteria Reagent (Qiagen, Cat. No. 76506), pelleted by centrifugation, and frozen at −80°C until RNA extraction. Total RNA was isolated as previously described (Schuster et al., 2003) using sonication and column-based purification (RNeasy Mini Kit, Qiagen, Cat. No. 74106), followed by treatment with DNase I (RNAse-free, New England Biolabs, Cat. No. M0303S), and RNeasy-based purification. Total RNA was subjected to rRNA-depletion using the Ribo-Zero™ protocol (Illumina Inc.), followed by cDNA synthesis and indexed, stranded library preparation using the WaferGen protocol on the robotic Apollo instrument (WaferGen Bio-systems Inc.). All 30 sample libraries were then pooled and evenly multiplexed into a single lane of paired-end 2 × 100 bp sequencing on the HiSeq3000 instrument (Illumina Inc.). cDNA libraries were prepared and sequenced at the Center for Genome Research and Biocomputing at Oregon State University (Corvallis, Oregon, USA). Sequences were separated according to index and filtered of contaminating adapter content bioinformatically. Raw .FASTQ files (containing sequence “reads”) were inspected for general quality (per base sequence quality >Q28) and sequence contamination using FastQC (http://www.bioinformatics.babraham.ac.uk), confirming no further pre-processing was necessary. The transcriptome data discussed in this publication have been deposited in NCBI's Gene Expression Omnibus (Edgar et al., 2002) and are freely accessible through GEO Series accession number GSE107758 (https://www.ncbi.nlm.nih.gov/geo/query/acc.cgi?acc=GSE107758).

### Transcriptome data analysis

We used the Burrows-Wheeler aligner BWA-MEM (Li and Durbin, 2010) to map processed reads to the *P. aeruginosa* PAO1 reference genome ORFs (PAO1_107; available at http://www.pseudomonas.com) (Winsor et al., 2016), followed by duplicate removal and count matrix generation in SAMtools with default parameters (Li et al., 2009). rRNA, tRNA, and tmRNA ORFs (http://www.pseudomonas.com) were manually removed yielding a count matrix of 5622 genes × 30 samples that was then loaded into the RStudio statistical environment (https://www.rstudio.com). Differential expression analysis was carried out using the DESeq2 package under standard settings using each strain-growth phase combination as a factor level (Love et al., 2014). Hypothesis testing was carried out in DESeq2 using the Benjamini Hochberg adjustment for multiple comparisons and a false-discovery rate (FDR) α = 0.05 with no high or low log_2_fold-change limits. Functional annotations were assigned using the most recent list of 22 predicted classes produced using publicly available PAO1 COG mappings (http://www.pseudomonas.com). Absolute expression comparisons were made using the regularized log transformation (rlog) in DESeq2 (Love et al., 2014). Data were visualized using the Heatmapper webtool (Babicki et al., 2016), ClustVis webtool (Metsalu and Vilo, 2015) and ggplot package in RStudio (Wickham, 2009).

## Results

### *lasB* promoter activity among anti-activator mutants

**Figure 2:**
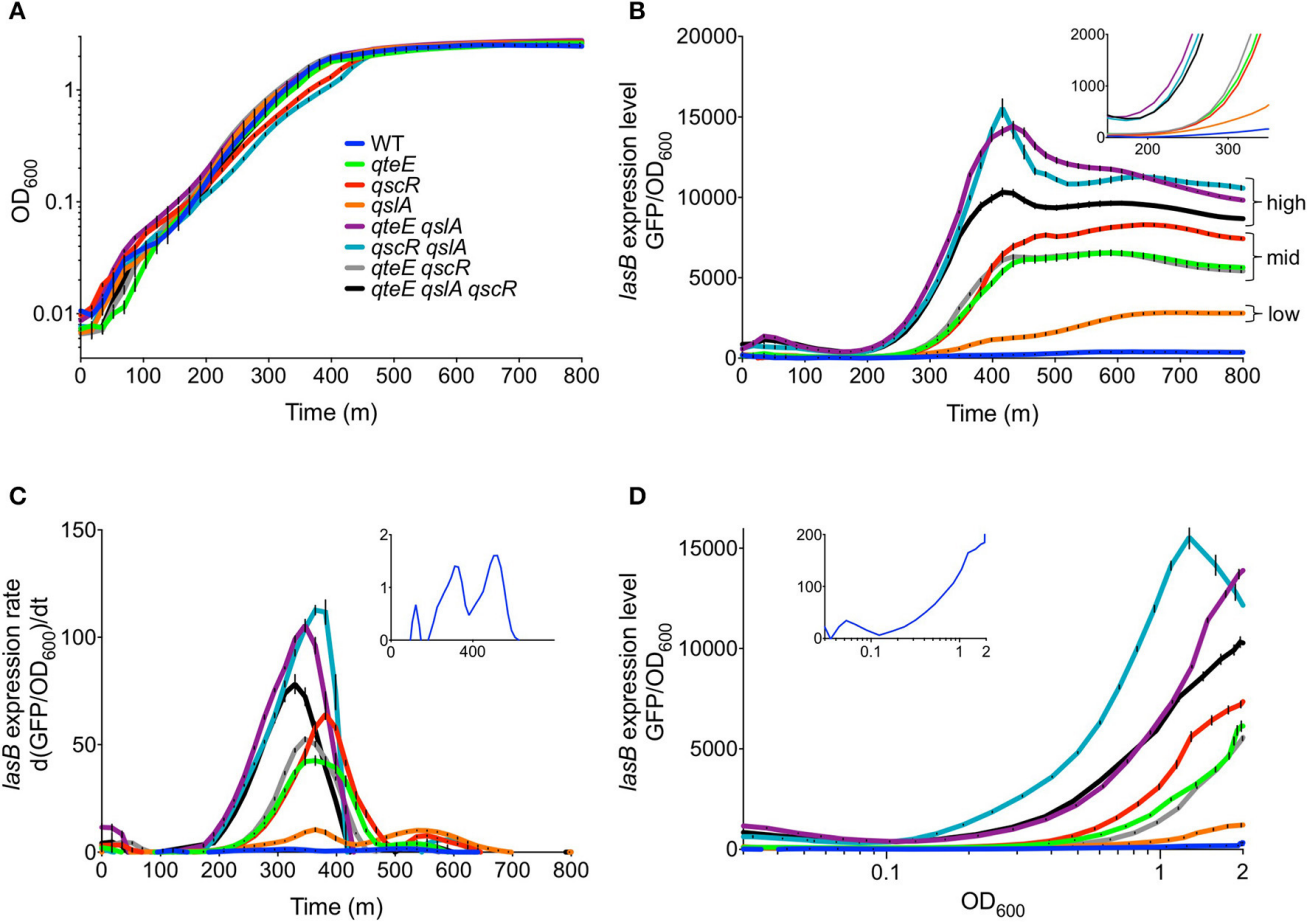
Effects of anti-activator gene deletion on *P*_*lasB*_-*gfp* expression kinetics**. (A)** Growth of strains in CAA medium. **(B)** *P*_*lasB*_-*gfp* expression levels vs. time. Expression levels are normalized to OD_600_. Inset has reduced *x*- and *y*-axes to emphasize expression timing. **(C)** *P*_*lasB*_-*gfp* expression rates vs. time, showing time derivatives of OD-normalized expression levels. Inset has a reduced *y*-axis to emphasize expression rate peaks in the wild-type. **(D)** OD-normalized *P*_*lasB*_-*gfp* expression levels vs. cell density. Inset has a reduced *y*-axis to emphasize the wild-type expression profile. In all panels, values represent means of three biological replicates. Error bars indicate s.e.m. (*n* = 3).

The effects of individual *qteE, qscR*, or *qslA* gene deletions on the induction of QS target genes have been examined by different research groups (Chugani et al., 2001; Siehnel et al., 2010; Fan et al., 2013; Gupta and Schuster, 2013), but a direct comparison of their individual effects and the effects of multiple deletions on timing and magnitude of QS expression has not been made. We assembled a set of anti-activator-null strains of PAO1 representing each possible combination of anti-activator-null alleles (7 mutants total; see Table 1 for a comprehensive list of strains and plasmids used in this study) to allow comparisons of anti-activator effects. *P. aeruginosa* LasB-elastase is a well-described Las- and Rhl-responsive proteolytic virulence factor, making *lasB* promoter activity an appropriate proxy for QS gene induction in this context (Pearson et al., 1997; Schuster et al., 2003, 2004b). We recorded *lasB* promoter activity through utilization of an established plasmid-borne *P*_*lasB*_-*gfp* transcriptional reporter, which shows QS-dependent expression under culture conditions identical to those employed here (Gupta and Schuster, 2013). We evaluated accumulation of *P*_*lasB*_-*gfp*-derived fluorescence during growth of the wild-type and our set of anti-activator mutants (Figure 2). All strains showed similar growth in CAA medium, with minor growth defects observed in the *qscR* and *qscR qslA* mutants as strains approached the end of log phase (Figure 2A). From these data, we also calculated specific expression rates (Figure 2C). The *qteE* and *qscR* single mutants showed 30- and 15-fold increases in maximum expression levels and rates (Figures 2B,C) compared to the wild-type. The *qslA* mutant only showed increases of roughly 7-fold in expression levels and rates. The *qteE qscR* double mutant registered values nearly identical to mutants harboring just a single one of these mutations, indicating a lack of additivity with these two anti-activators. However, with any other combination of deleted anti-activator alleles *(qteE qslA* or *qteE qscR), P*_*lasB*_-*gfp* induction was increased further in both total expression levels and rates, with the triple anti-activator mutant showing a slightly lower increase (Figures 2B,C). The timing of induction only changed for our three strains showing the highest expression levels. *qscR qslA, qteE qslA*, and *qteE qslA qscR* mutants all showed *P*_*lasB*_-*gfp*-activation and rapid increases in expression rates starting at approximately 200 min, with all other mutants and the wild type showing activation occurring roughly 60–120 min later (Figure 2B, inset). Induction kinetics were generally similar when plotted vs. cell density (Figure 2D) instead of time (Figure 2B). Owing to their slight growth differences in late log phase, the *qscR* and *qscR qslA* mutants achieved relatively higher expression levels at equal cell densities. In summary, all measurements of overall mutant *P*_*lasB*_-*gfp* expression levels and rates were higher in the mutants than the wild-type, with three groups emerging with similar profiles: the *qslA* mutant with the smallest increase in expression (“low”), the *qteE, qscR*, and *qteE qscR* mutants with moderate increases (“mid”), and the *qscR qslA, qteE qslA*, and *qteE qslA qscR* mutants showing the highest expression (“high”) (Figure 2B).

A closer look at the expression rates of the wild-type revealed a biphasic pattern with two distinct peaks (Figure 2C, inset) at approximately 310 and 500 min, likely corresponding to the sequential induction of the Las and Rhl QS systems, respectively (Gupta and Schuster, 2013). In all mutants except *qslA*, we observed a general shift in the relative expression rates to favor much higher expression rates during the initial, presumably Las-dependent, rate peak. This induction pattern suggests that the anti-activator proteins tested here primarily target LasR rather than RhlR.

### Elastase, pyocyanin, and LasR levels in anti-activator mutants

**Figure 3:**
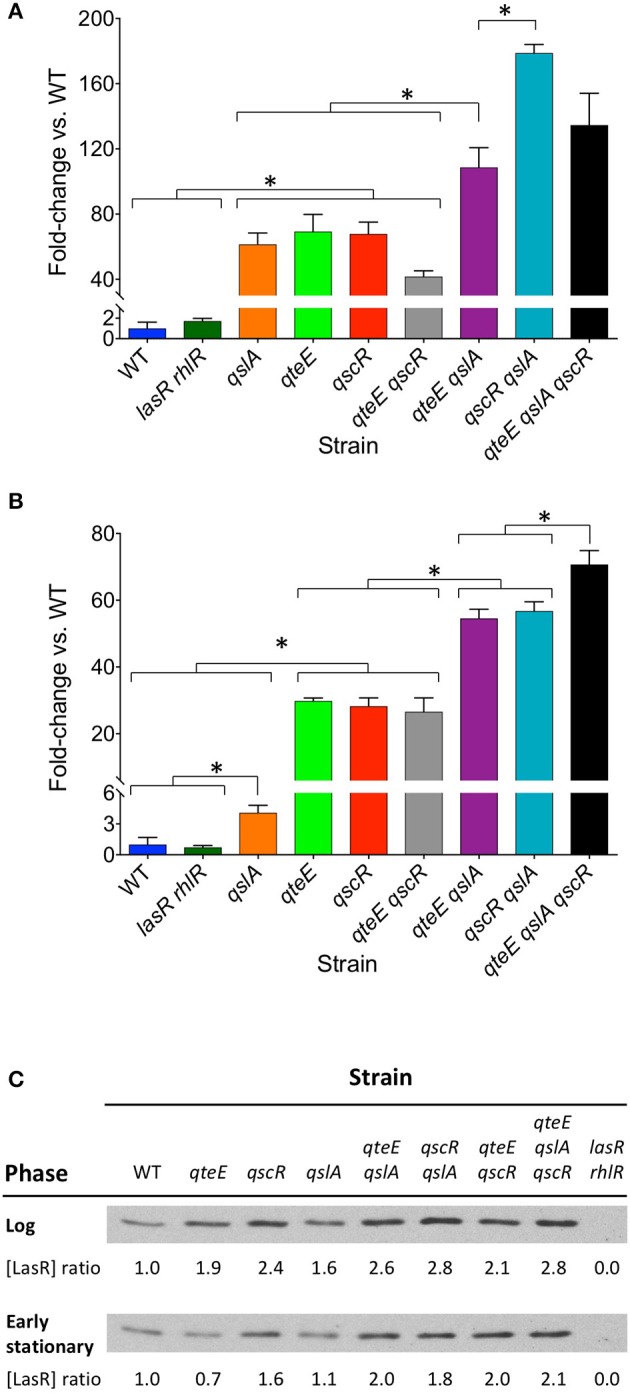
Effects of anti-activator gene deletion on QS phenotypes**. (A)** Elastase activity. **(B)** Pyocyanin production. Each assay was performed separately after 18 h of growth in CAA medium (stationary phase, OD_600_ = 1.9–2.3). In both panels, values represent means of three biological replicates, and *y*-axes are split to allow visualization of the lowest values. Error bars indicate s.e.m. (*n* = 3). Bars are grouped for clarity. Significant differences (^*^) in selected individual pairwise comparisons were determined using a two-tailed *T*-test (α = 0.05). **(C)** LasR Western analysis. Equal amounts of protein (5 μg) were added to each well. Log phase and early stationary phase blot bands are from separate gels, and therefore may not be directly comparable. Blots pictured are representative of replicate experiments. Relative LasR levels within each growth phase were quantified by densitometric analysis and are presented as ratios relative to wild-type.

To support our observations of differing effects of some anti-activator combinations on *P*_*lasB*_-*gfp* expression, we examined two characteristic QS-dependent virulence phenotypes in *P. aeruginosa*, pyocyanin, and elastase production, in CAA stationary-phase cultures. Levels of pyocyanin and elastase activity were roughly equivalent between the wild type and the *lasR rhlR* mutant (Figures 3A,B; not significantly different, α = 0.05), reflecting the generally lower level of secretion in this medium compared with LB, where differences between the two strains are discernable (Siehnel et al., 2010). However, elastase and pyocyanin production levels in the different anti-activator mutant combinations are significantly higher than wild type and generally mirrored those observed with *P*_*lasB*_-*gfp* fusions. Single mutants produced intermediate levels, and the double mutants harboring a *qslA* deletion as well as the triple mutant produced the most. The *qteE, qscR* double mutant grouped together with most single mutants. Notably, the *qslA* mutant produced as much elastase as the other single mutants, but significantly less pyocyanin.

We reasoned that the levels of *lasB* expression and virulence factor production in each strain could potentially be explained by the relative concentrations of LasR within cells, which should increase in the absence of anti-activators. To test this idea, we examined the relative LasR protein levels in the soluble fraction of cell lysates using densitometric analysis of Western blots (Figure 3C). In log phase (OD_600_ = 0.2), LasR ratios between strains reflect the dynamics of early induction seen in Figures 2B,C. Mutants designated as “high,” “mid,” or “low” showed soluble LasR ratios relative to wild-type of ≥2.6, 1.9–2.4, and 1.6, respectively. LasR ratios were overall lower in early stationary phase (OD_600_ = 1.6). With the exception of the *qteE* and *qslA* single mutants, relationships among “high,” “mid,” and “low” designated mutants and wild-type were generally similar to those observed in log phase. The *qteE* and *qslA* single mutants showed LasR levels roughly equivalent to wild-type in early stationary phase (ratios of 0.7 and 1.1, respectively).

### Identification of QteE and QslA regulons

**Figure 4:**
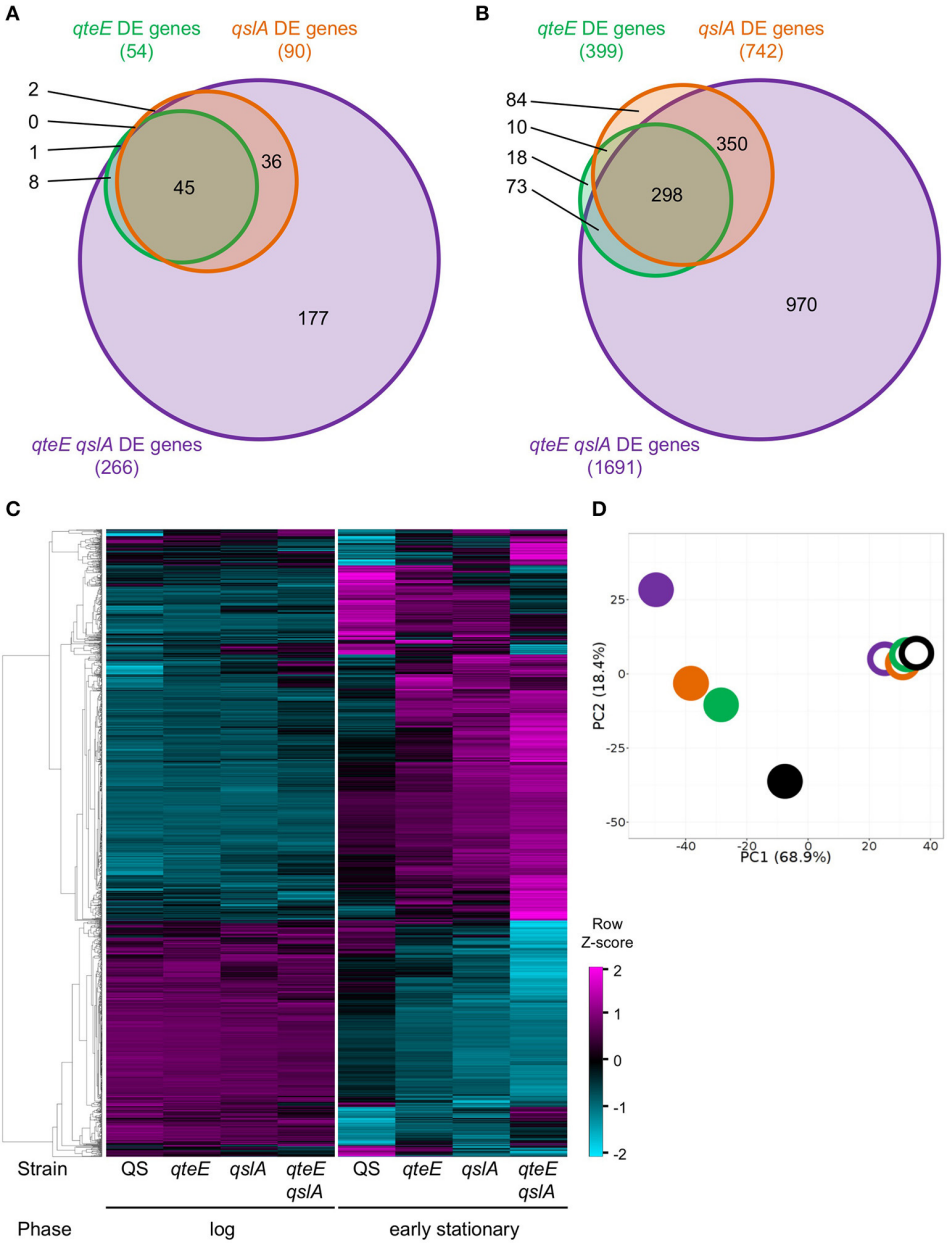
Comparison of differentially expressed (DE) genes among anti-activator mutants. **(A)** DE genes in log phase. **(B)** DE genes in early stationary phase. DE genes were determined in DESeq2 using three biological replicates (false discovery rate α = 0.05, *n* = 3). Venn diagram variables are roughly scaled to reflect quantities in order to visualize nesting. **(C)** Absolute expression. Expression levels were calculated as the Z-score for individual samples among rows of both log and early stationary phase regularized log (rlog) values generated in DESeq2 using three biological replicates (*n* = 3). Rows selected represent all DE genes from anti-activator mutant comparisons (1803 genes total) and are clustered by average linkage using the Pearson correlation. **(D)** Principal component analysis (PCA) of absolute expression results depicted in **(C)**. Unit variance scaling is applied to rows; singular value decomposition with imputation is used to calculate principal components. *x* and *y* axis show principal component 1 and principal component 2 that explain 68 and 18% of the total variance, respectively (*n* = 8 data points). Open symbols, log phase; filled symbols, early stationary phase. Black, wild-type; green, *qteE*; orange, *qslA*; purple, *qteE qslA*.

Having demonstrated anti-activator effects on QS phenotypes and LasR stability in addition to promoter activity dynamics of a QS gene, we sought to uncover the global scope of QS anti-activators with transcriptome profiling. We focused on *qteE* and *qslA* mutants, alone and in combination, which produced the most consistent additive effects on gene expression based on our analysis above, and which had not been previously profiled. Using an RNA-seq-based transcriptomics approach, we identified all genes that were differentially expressed (DE, α = 0.05) when mutants (*qteE, qslA, qteE, qslA*) were compared to wild-type in both log and early stationary phase. Both single anti-activator mutants showed differential expression of hundreds of genes, with the *qteE* mutant showing 415 differentially expressed genes, and the *qslA* mutant showing roughly double that quantity at 770 genes (Table 2, see Table S1 for all DE genes). We observed a synergistic effect of deletion of both anti-activators with the *qteE qslA* mutant differentially expressing a total of 1797 genes, corresponding to roughly 31 percent of all *P. aeruginosa* genes. Consistent with a common functional role, the three different gene sets showed substantial overlap in both log and early stationary phase (Figure 4), and most genes affected by anti-activator gene deletion showed activation (Table 2). Sets of anti-activator-affected genes were effectively nested; regardless of growth phase, more than 75 percent of *qteE*-affected genes were also *qslA*-affected, and more than 85 percent of *qslA*-affected genes were also affected in the double mutant (Figures 4A,B). This finding is consistent with anti-activators functioning by sequestering transcriptional activators to different degrees, with the *qteE qslA*-affected gene set encompassing both single-mutant gene sets.

**Table 2:** Differentially expressed genes.

**Strain comparison**	**Log**			**Early stationary**			**All DE genes^a^**		
	**Induced**	**Repressed**	**Total**	**Induced**	**Repressed**	**Total**	**Induced**	**Repressed**	**Total**
WT vs. *lasR rhlR*	1	–	1	79	59	138	79	59	138
*qteE* vs. WT	52	2	54	298	101	399	312	103	415
*qslA* vs. WT	82	8	90	477	265	742	500	270	770
*qteE qslA* vs. WT	214	52	266	934	757	1691	999	798	1797

a *All DE genes values were produced by adding Log and Early stationary gene lists and removing duplicates. DE, differentially expressed; WT, wild-type*.

In addition, numerous genes were repressed by anti-activator gene deletion (Table 2). Presumably, these genes are either also regulated by QS (indirectly through LasR or RhlR-dependent activation of a transcriptional repressor or through another, as yet unknown mechanism), or they are regulated independently of QS and the presumed R-protein sequestration mechanism.

Next, we determined whether the size of the respective anti-activator regulons is reflected in the magnitude of gene expression. We hierarchically clustered the absolute expression patterns of all genes differentially expressed in the anti-activator mutants (1803 genes total; Figure 4C). Clustering produced a clear pattern separating most genes induced by anti-activator deletion from those that were repressed, with large expression differences between log and stationary phase. The expression pattern of all induced genes shows successively higher expression levels according to regulon size, again mirroring the nested effect seen in our regulon comparisons (Figures 4A,B). Repressed genes also showed a similar pattern of successively lower expression levels according to regulon size (Figure 4C). Both patterns are more nuanced in stationary than in log phase. To examine these trends further, we used principle component analysis (PCA) to generalize our observations of differences among our collection of strain-growth phase expression profiles. PCA analysis of anti-activator regulons in log and stationary phase generally supports these observations, with stationary phase measurements of anti-activator mutants driving nearly 69% of the variability in our dataset (Figure 4D). Stationary phase profiles clearly segregated into three groups representing the strain with functional anti-activators (wild-type), strains lacking a single anti-activator, and the *qteE qslA* mutant largely separated from all other strains. Additional variability up to 18% of the total observed also appears to be driven by individual anti-activator mutants, with the greatest difference observed between the *qteE qslA* double mutant and the wild-type.

### Identification of a QS regulon

**Figure 5:**
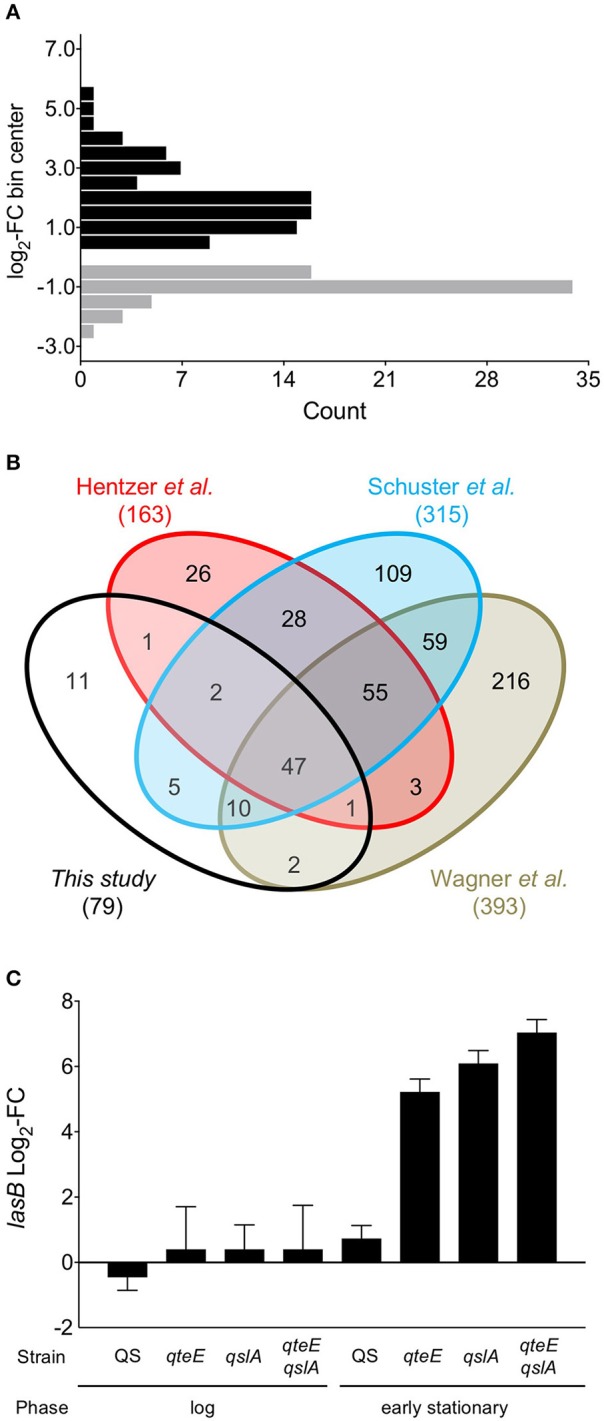
A QS-controlled regulon**. (A)** Histogram of genes differentially expressed in a wild-type vs. *lasR rhlR* mutant comparison from early stationary phase cultures grown in CAA medium. Differentially expressed (DE) genes activated (black bars) and repressed (gray bars) in the comparison were determined in DESeq2 using three biological replicates (false discovery rate α = 0.05, *n* = 3). FC, fold-change. Values were binned to span defined intervals of 0.5 × log_2_-FC, and bins were positioned over interval centers in the histogram. **(B)** Comparison of QS regulons among previous microarray results (Hentzer et al., 2003; Schuster et al., 2003; Wagner et al., 2003) and the present study. Venn diagrams are not scaled to gene number. **(C)** Comparison of *lasB* (PA3724) fold-change in the RNA-seq experiment. “QS” fold-change represents results from the wild-type vs. *lasR rhlR* comparison; all others represent results of individual comparisons with each strain vs. the wild-type.

Next, to evaluate the relationship between our anti-activator-affected genes and QS, we determined a QS regulon for the wild-type strain under our culture conditions. We identified all differentially expressed (DE, α = 0.05) genes between our wild-type strain and an isogenic *lasR rhlR* mutant in both log and early stationary phase. Based on previous studies, we expected many more DE genes in early stationary phase than in the log phase comparison as these growth phases represent quorum “ON” and “OFF” states, respectively (Schuster et al., 2003; Wagner et al., 2003). We found 138 differentially expressed genes in early stationary phase between the wildtype and *lasR rhlR* mutant, including 79 quorum-activated and 59 quorum-repressed genes (Figure 5A, Tables 3, 4). The only DE gene detected in our log phase comparison was *lasR* itself, supporting the design of our log phase (QS “OFF”) vs. early stationary phase (QS “ON”) comparison. As genes activated in the quorum regulon are consistent with the established function of LasR and RhlR as transcriptional activators (Whiteley et al., 1999; Kiratisin et al., 2002; Hentzer et al., 2003; Schuster et al., 2003; Schuster and Greenberg, 2007) and the established function of anti-activators as factors for R-protein sequestration and destabilization (Piper and Farrand, 2000; Siehnel et al., 2010; Fan et al., 2013), we focused our subsequent analysis on activated genes.

**Table 3:** Quorum-activated genes.

**Locus tag^a^**	**Name^a^**	**Annotation^a^**	**Early stationary phase fold change^b^**			
			**QS**	***qteE***	***qslA***	***qteE qslA***
PA0026	*plcB*	phospholipase C, PlcB	2.5	NC	2.3	2.1
PA0027		hypothetical protein	2.2	1.8	**3.1**	**2.4**
PA0052		hypothetical protein	2.3	4.6	9.6	14.9
PA0143	*nuh*	purine nucleosidase Nuh	1.9	**1.9**	**3.8**	**3.8**
PA0178		probable two-component sensor	2.4	NC	NC	1.7
PA0524	*norB*	nitric-oxide reductase subunit B	7.8	NC	NC	−10.7
PA0572		hypothetical protein	3.6	2.9	**4.5**	**2.8**
PA1130	*rhlC*	rhamnosyltransferase 2	4.3	5.4	5.6	**6.8**
PA1131		probable major facilitator superfamily (MFS) transporter	11.0	6.1	5.3	**7.0**
PA1246	*aprD*	alkaline protease secretion protein AprD	3.3	NC	NC	**2.5**
PA1248	*aprF*	Alkaline protease secretion outer membrane protein AprF precursor	2.6	NC	NC	**3.0**
PA1249	*aprA*	alkaline metalloproteinase precursor	3.6	3.6	4.5	7.0
PA1250	*aprI*	alkaline proteinase inhibitor AprI	3.7	**3.1**	**3.6**	**3.5**
PA1251		probable chemotaxis transducer	3.3	**2.5**	**3.3**	**3.7**
PA1430	*lasR*	transcriptional regulator LasR	40.7^*^	1.7	**2.5**	**3.6**
PA1431	*rsaL*	regulatory protein RsaL	6.1	**1.9**	**3.1**	4.1
PA1432	*lasI*	autoinducer synthesis protein LasI	28.0	**NC**	−**2.5**	−1.6
PA1433		conserved hypothetical protein	1.6	**NC**	**NC**	NC
PA1656	*hsiA2*	HsiA2	3.5	**12.0**	**8.0**	**5.8**
PA1663	*sfa2*	Sfa2	2.3	9.2	4.5	**2.8**
PA1668	*dotU2*	DotU2	1.8	8.6	**3.6**	**2.4**
PA1784		hypothetical protein	2.3	3.5	7.2	13.4
PA1869		probable acyl carrier protein	3.5	**25.1**	**20.5**	**23.6**
PA1871	*lasA*	LasA protease precursor	3.4	22.9	22.9	36.6
PA1893		hypothetical protein	2.5	**1.6**	NC	**2.3**
PA1894		hypothetical protein	3.9	**2.4**	**NC**	**3.2**
PA1895		hypothetical protein	2.3	**2.1**	NC	**2.9**
PA1896		hypothetical protein	2.4	**1.7**	NC	**2.9**
PA1897		hypothetical protein	2.5	**2.3**	**NC**	**3.2**
PA2076		probable transcriptional regulator	1.8	**1.6**	**2.4**	**2.2**
PA2080	*kynU*	kynureninase KynU	1.7	**1.7**	**2.8**	**2.1**
PA2081	*kynB*	kynurenine formamidase, KynB	2.5	**1.9**	**2.7**	**2.5**
PA2193	*hcnA*	hydrogen cyanide synthase HcnA	4.9	12.1	19.3	**7.4**
PA2194	*hcnB*	hydrogen cyanide synthase HcnB	5.4	12.7	**19.0**	**7.3**
PA2195	*hcnC*	hydrogen cyanide synthase HcnC	3.8	13.8	20.8	**8.9**
PA2301		hypothetical protein	4.0	2.7	3.0	3.5
PA2302	*ambE*	AmbE	18.9	**3.0**	**3.9**	**4.2**
PA2303	*ambD*	AmbD	25.6	**3.0**	**3.7**	**4.3**
PA2304	*ambC*	AmbC	13.3	**2.9**	**3.3**	**4.3**
PA2305	*ambB*	AmbB	12.2	**3.3**	**4.8**	**4.8**
PA2423		hypothetical protein	3.1	**NC**	**3.1**	**3.9**
PA2587	*pqsH*	probable FAD-dependent monooxygenase	8.1	**3.9**	**4.3**	**5.6**
PA2588		probable transcriptional regulator	1.9	**7.9**	**7.0**	**24.4**
PA2591	*vqsR*	VqsR	7.1	**2.4**	**3.0**	**2.3**
PA2592		probable periplasmic spermidine/putrescine-binding protein (*potF5*)	3.7	**5.2**	**4.1**	**4.7**
PA2607		conserved hypothetical protein	1.6	NC	NC	NC
PA2608		conserved hypothetical protein (*yccK*)	1.5	NC	NC	NC
PA2939		probable aminopeptidase (*pepB*)	2.7	4.1	9.1	11.5
PA2949		probable lipase	1.4	NC	NC	NC
PA3326	*clpP2*	ClpP2	2.5	7.2	7.3	**7.0**
PA3327		probable non-ribosomal peptide synthetase	3.3	16.1	8.1	**3.5**
PA3328		probable FAD-dependent monooxygenase	4.5	21.9	12.4	**5.4**
PA3329		hypothetical protein	3.6	25.1	13.9	**5.9**
PA3330		probable short chain dehydrogenase	4.1	18.3	11.1	**4.4**
PA3331		cytochrome P450	3.5	20.3	11.6	**4.8**
PA3332		conserved hypothetical protein	3.3	23.4	13.4	**4.8**
PA3333	*fabH2*	3-oxoacyl-[acyl-carrier-protein] synthase III	4.4	22.9	11.9	**4.0**
PA3336		probable major facilitator superfamily (MFS) transporter	2.6	18.5	10.0	**41.0**
PA3346		two-component response regulator	1.7	NC	2.0	**2.8**
PA3391	*nosR*	regulatory protein NosR	8.6	NC	NC	−21.7
PA3392	*nosZ*	nitrous-oxide reductase precursor	10.7	NC	NC	−13.5
PA3476	*rhlI*	autoinducer synthesis protein RhlI	10.5	**4.1**	**2.9**	**4.3**
PA3477	*rhlR*	transcriptional regulator RhlR	7.2	**2.3**	**3.0**	**4.0**
PA3479	*rhlA*	rhamnosyltransferase chain A	2.2	36.7	24.3	**74.0**
PA3535		probable serine protease (*eprS*)	2.8	**2.2**	**5.2**	**6.0**
PA3615		hypothetical protein	1.6	NC	NC	−1.5
PA3904		hypothetical protein	15.0	**2.6**	**3.3**	**2.5**
PA3905		hypothetical protein	10.5	**2.4**	**3.0**	**1.6**
PA3906		hypothetical protein	17.4	**NC**	**3.1**	**NC**
PA3907		hypothetical protein	8.4	**2.7**	**4.1**	**NC**
PA3908		hypothetical protein	5.8	**2.9**	**4.3**	**2.4**
PA4117	*bphP*	bacterial phytochrome, BphP	1.8	**1.7**	**2.9**	**4.3**
PA4190	*pqsL*	probable FAD-dependent monooxygenase	2.5	**NC**	**NC**	**NC**
PA4594		probable ATP-binding component of ABC transporter	1.9	NC	2.1	2.6
PA4677		hypothetical protein	1.8	3.7	**3.1**	**3.6**
PA4778	*cueR*	CueR (*ybbI*)	1.8	**2.4**	**3.5**	**5.0**
PA4869		hypothetical protein	1.7	**NC**	**2.4**	**2.7**
PA4955		hypothetical protein	1.6	NC	NC	NC
PA5255	*algQ*	Alginate regulatory protein AlgQ (*algR2*)	1.5	NC	NC	NC

a *Locus tags, gene names, and gene annotations from the Pseudomonas Genome Database (http://www.pseudomonas.com)*.

b *QS represents the WT vs. lasR rhlR comparison, while all anti-activator mutant comparisons are vs. the wild-type. BOLD denotes genes of the quorum-activated regulon in early stationary phase also differentially expressed in log phase. Negative values indicate repression, positive values indicate activation. NC, no change*.

* *This fold change estimate represents native expression of lasR in the wild-type vs. no expression in the lasR rhlR mutant*.

**Table 4:** Quorum-repressed genes.

**Locus tag^a^**	**Name^a^**	**Annotation^a^**	**Early stationary phase fold change^b^**			
			**QS**	***qteE***	***qslA***	***qteE qslA***
PA0045		hypothetical protein	−2.2	NC	−2.9	−3.3
PA0047		hypothetical protein	−2.3	NC	−1.9	−2.3
PA0592	*ksgA*	rRNA (adenine-N6,N6)-dimethyltransferase	−1.6	NC	NC	−1.4
PA0944	*purN*	phosphoribosylaminoimidazole synthetase	−1.8	NC	NC	NC
PA1302		probable heme utilization protein precursor (*hxuC*)	−2.1	NC	NC	NC
PA1303		signal peptidase	−2.4	NC	NC	NC
PA1542		hypothetical protein	−1.8	NC	1.7	1.9
PA1580	*gltA*	citrate synthase (*cisY*)	−1.6	NC	NC	NC
PA1595		hypothetical protein	−1.9	NC	NC	NC
PA1757	*thrH*	homoserine kinase	−2.0	NC	NC	NC
PA1791		hypothetical protein	−1.9	NC	−**2.1**	−3.1
PA2583		probable sensor/response regulator hybrid	−1.7	NC	NC	NC
PA2665	*fhpR*	Transcriptional activator of P. aeruginosa flavohemoglobin, FhpR (*ygaA*)	−1.7	NC	NC	NC
PA2770		hypothetical protein	−1.7	NC	NC	2.3
PA2780	*bswR*	bacterial swarming regulator BswR	−1.5	**NC**	**NC**	**NC**
PA2930		probable transcriptional regulator	−2.4	NC	NC	NC
PA2950	*pfm*	proton motive force protein, PMF	−1.6	NC	NC	NC
PA2964	*pabC*	4-amino-4-deoxychorismate lyase	−1.5	NC	NC	NC
PA2970	*rpmF*	50S ribosomal protein L32	−2.1	NC	NC	NC
PA2998	*nqrB*	Na+-translocating NADH:ubiquinone oxidoreductase subunit Nrq2	−1.8	NC	NC	NC
PA3079		hypothetical protein	−1.9	NC	NC	NC
PA3111	*folC*	folylpolyglutamate synthetase	−1.6	NC	NC	NC
PA3174		probable transcriptional regulator	−1.9	−2.3	−2.7	−**3.2**
PA3268		probable TonB–dependent receptor	−3.4	NC	NC	**NC**
PA3284		hypothetical protein	−3.3	−5.5	−7.4	−8.8
PA3362		hypothetical protein (*amiS*)	−2.3	8.0	12.4	7.3
PA3473		hypothetical protein	−1.7	NC	NC	NC
PA3609	*potC*	polyamine transport protein PotC	−2.0	NC	NC	NC
PA3820	*secF*	secretion protein SecF	−2.5	NC	NC	NC
PA3823	*tgt*	queuine tRNA-ribosyltransferase	−1.8	NC	NC	−1.8
PA3827	*lptG*	Lipopolysaccharide export system permease protein LptG (*yjgQ*)	−1.5	NC	NC	NC
PA3979		hypothetical protein	−1.6	NC	NC	NC
PA4045		conserved hypothetical protein (*btuF*; *yadT*)	−1.7	NC	NC	NC
PA4046		hypothetical protein	−1.5	NC	NC	NC
PA4375	*mexW*	Resistance-Nodulation-Cell Division (RND) multidrug efflux transporter MexW	−1.7	NC	NC	NC
PA4479	*mreD*	rod shape-determining protein MreD	−2.8	NC	NC	NC
PA4519	*speC*	ornithine decarboxylase	−1.8	1.9	2	3.2
PA4562		conserved hypothetical protein (*mviN*)	−1.7	NC	NC	NC
PA4569	*ispB*	octaprenyl-diphosphate synthase (*cel*)	−1.8	NC	NC	−1.9
PA4628	*lysP*	lysine-specific permease	−1.7	NC	NC	NC
PA4630		hypothetical protein	−2.0	−1.6	−2.6	−2.5
PA4672		peptidyl-tRNA hydrolase (*pth*)	−2.1	NC	NC	NC
PA4757		conserved hypothetical protein (*yeaS*)	−1.5	NC	NC	−1.4
PA4840		conserved hypothetical protein (*yciH*)	−1.6	NC	NC	NC
PA5072		probable chemotaxis transducer	−1.5	NC	NC	NC
PA5081		hypothetical protein	−2.0	−1.8	NC	−1.5
PA5117	*typA*	regulatory protein TypA (*bipA*)	−1.7	NC	NC	NC
PA5139		hypothetical protein	−2.2	NC	NC	−3.3
PA5156		hypothetical protein	−1.8	NC	NC	NC
PA5167	*dctP*	DctP	−3.9	NC	NC	NC
PA5168	*dctQ*	DctQ	−4.3	NC	NC	NC
PA5169	*dctM*	DctM	−4.9	NC	NC	NC
PA5194		hypothetical protein	−1.7	NC	NC	NC
PA5250		conserved hypothetical protein	−1.7	NC	NC	NC
PA5251		hypothetical protein	−1.7	NC	NC	NC
PA5320	*coaC*	Phosphopantothenoylcysteine synthase/(R)-4′-phospho-N-pantothenoylcysteine decarboxylase (*coaB*; *coaBCI*; *dfp*)	−1.4	NC	NC	−1.3
PA5361	*phoR*	two-component sensor PhoR	−1.6	NC	NC	NC
PA5492		conserved hypothetical protein (*ysxC*; *yihA*)	−1.9	NC	NC	NC
PA5560	*atpB*	ATP synthase A chain (*papD, uncB*)	−1.8	NC	NC	−2.4

a *Locus tags, gene names, and gene annotations from the Pseudomonas Genome Database (http://www.pseudomonas.com)*.

b *QS represents the WT vs. lasR rhlR comparison, while all anti-activator mutant comparisons are vs. the wild-type. BOLD denotes genes of the quorum-repressed regulon in early stationary phase also differentially expressed in log phase. Negative values indicate repression, positive values indicate activation. NC, no change*.

We compared our quorum-activated genes with those published previously using microarrays (Hentzer et al., 2003; Schuster et al., 2003; Wagner et al., 2003). While media choice, growth phases tested, and strain backgrounds vary among these studies, previous comparisons suggest a core QS regulon in *P. aeruginosa* that may be activated in most strains (Schuster and Greenberg, 2006). In our 4-way comparison we found 68 of our 79 genes were shared with at least one previous study, and a core regulon of 47 quorum-activated genes is shared among all 4 studies (Figure 5B). The large overlap of the quorum-activated regulon described here with those in previous microarray experiments validated our approach, as well as the general observation of a core QS-regulon among different *P. aeruginosa* strains and growth conditions (Chugani et al., 2012). The core QS-regulon determined here includes many well-studied targets of QS activation: *rhlA* (PA3479), encoding rhamnosyl transferase; *hcnABC* (PA2193-95), encoding hydrogen cyanide synthase, the *apr* cluster (PA1246-50), encoding alkaline protease; *rsaL* (PA1430), a transcriptional repressor of LasI; *rhlI* and *rhlR* (PA3476-7), encoding the Rhl QS machinery and *pepB* (PA2939), encoding the aminopeptidase PepB (Table 3).

We did not identify the *lasB* gene (PA3724) as differentially expressed in our QS regulon, which contrasts with the induction pattern of this quorum-activated gene based on *P*_*lasB*_-*gfp* expression analysis (Figure 2; Gupta and Schuster, 2013). In our previous study, *P*_*lasB*_-*gfp* expression in CAA medium was significantly higher in the wild type than in a *lasR* QS mutant in late log phase and throughout stationary phase (Gupta and Schuster, 2013). Our transcriptome sampling time in early stationary phase was guided in part by these *lasB* expression data, although we recognize that accumulation of stable GFP expressed from a multi-copy plasmid likely exaggerates gene expression changes obtained by transcriptomics. In addition, our sampling scheme was guided by a previous microarray study in LB medium (Schuster and Greenberg, 2007), where the vast majority of QS genes showed high induction in early stationary phase. In comparison with LB, CAA medium not only produces lower induction levels of QS-controlled genes but also produces a smaller QS regulon overall (not considering differences in statistical analysis). This may be, at least in part, a result of the generally lower culture densities in CAA medium than in LB medium. We also observed relatively low levels of elastase activity in the wild-type strain used here (Figure 2B). The *lasB* expression values from our transcriptome dataset alone are in agreement with these phenotypic results. In the transcriptome results, the wild-type shows a modest increase in expression compared to the *lasR rhlR* mutant (2-fold, not significant, α = 0.05), while the presence of any anti-activator mutation drives *lasB* expression beyond 37-fold (*qteE*) and up to roughly 130-fold (*qslA*) (Figure 5C). Thus, sampling times and growth conditions likely explain the absence of *lasB* in our experimentally determined quorum-activated regulon.

### Deletion of *qteE* and *qslA* advance timing and increase magnitude of QS gene expression

**Figure 6:**
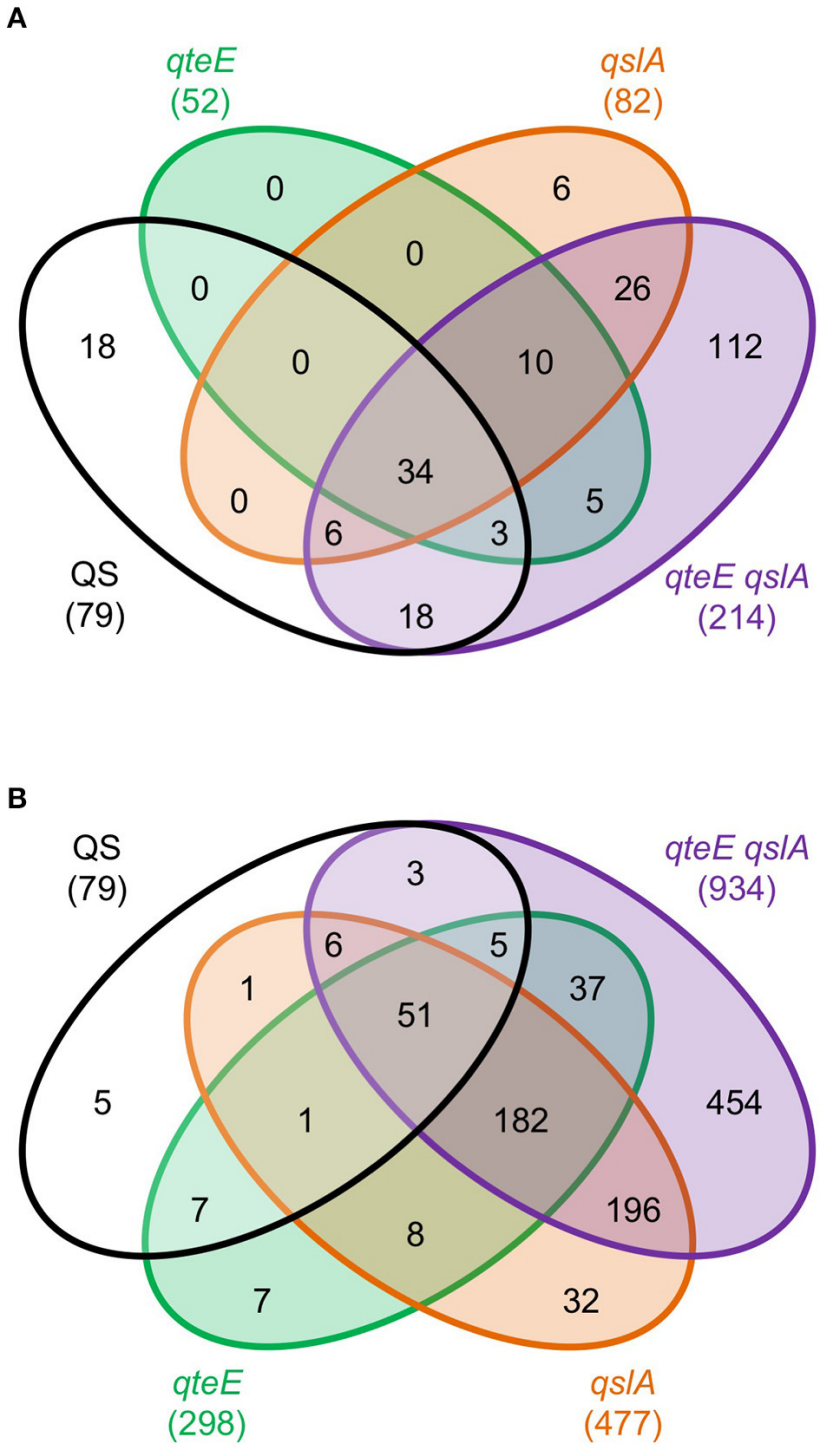
Overlap of induced genes in QS and anti-activator regulons**. (A)** Log phase anti-activator regulons and early stationary phase QS regulon. **(B)** Early stationary phase anti-activator regulons and early stationary phase QS regulon. For both panels: Differentially expressed (DE) genes were determined in DESeq2 (see Materials and Methods) using three biological replicates (false discovery rate α = 0.05, *n* = 3). Values represent only induced genes, and Venn diagrams are not scaled to gene number.

The quantity of differentially expressed genes in log phase was drastically higher in the anti-activator mutant/wild-type comparisons than in the wild-type/*lasR rhlR* mutant comparison (Table 2), so we reasoned that many of the former were genes from the quorum-activated regulon that exhibited advanced timing. To test this, we compared expression of genes induced in the *qteE, qslA*, and *qteE qslA* mutants in log phase with our quorum-activated regulon in early stationary phase. Genes listed in the quorum-activated regulon that are differentially expressed in anti-activator mutants in log phase can then be said to be the result of advancement of timing in the quorum threshold due to absence of QS anti-activation. The large majority of quorum-induced genes (61 of 79, 77%) in early stationary phase were advanced to log phase through deletion of *qteE, qslA*, or both (Figure 6A). In addition, the nested character of the anti-activator regulons, as mentioned above, was again apparent here. Both features reinforce the notion that QteE and QslA function by R-protein sequestration.

**Figure 7:**
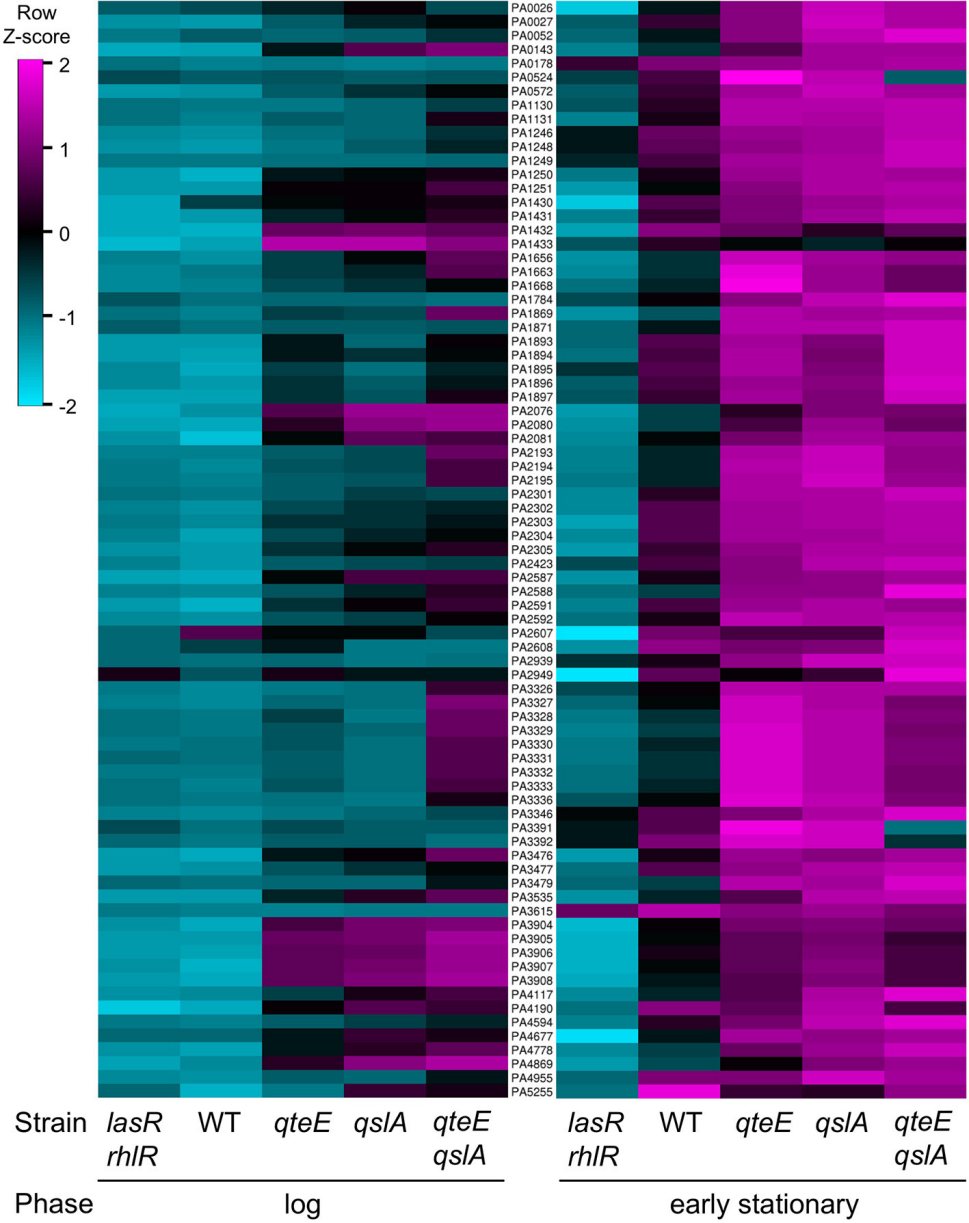
Absolute expression of the QS regulon. Absolute expression was calculated as the *Z*-score for individual samples among rows of both log and early stationary phase regularized log (rlog) values generated in DESeq2 (see Materials and Methods) using three biological replicates (*n* = 3). Rows selected represent only induced genes in the QS regulon and are ordered by locus tag (middle column) for reference. WT, wild-type.

We continued analysis of anti-activator effects through comparison of the 79 quorum-activated genes with anti-activator mutant gene expression in early stationary phase. In all, 74 of the 79 quorum-activated genes, or 93%, were differentially expressed by a mutant deficient in at least one anti-activator protein (Figure 6B). Fifty-one of those 74 were differentially expressed in all mutants tested. We then questioned whether absolute expression of the quorum-activated regulon as a whole differs among our anti-activator mutants, similar to the pattern observed in Figure 4C. Deletion of *qteE* or *qslA* appears to produce a pattern of increased absolute expression among quorum-activated genes in both log and early stationary phase (Figure 7). For several QS genes, loss of anti-activation shows a step-wise increase in absolute expression during log phase moving from *qteE* to *qslA* to the *qteE qslA* double mutant. These genes include: *nuh* (PA0143), encoding the purine nucleosidase Nuh; *rsaL* (PA1431); *kynU* (PA2080) encoding the kynureninase KynU; *cueR* (PA4778), encoding the copper toxicity transcriptional regulator CueR; and a cluster of relatively evenly expressed genes (PA3904-8) encoding hypothetical proteins. A select group of nitrate respiration genes (*norB*, PA0524; *nosR*, PA3391) in the QS regulon exhibited a nearly opposite pattern, showing maximal absolute expression in the *qteE* mutant, lower expression in the *qslA* mutant, and lowest absolute expression in the *qteE qslA* mutant. The expression of the QS circuitry genes *lasR, rhlR*, and *rhlI* was elevated in the absence of anti-activators (Table 3, Figure 7), which is expected due to positive auto-regulation (Pesci et al., 1997; Croda-Garcia et al., 2011). In contrast, the expression of the signal synthase gene *lasI* was decreased in anti-activator mutants, which can be explained by a concomitant increase in the expression of *rsaL*, encoding a transcriptional repressor of *lasI* (Rampioni et al., 2007).

While 93% of the genes in the QS-activated regulon were represented in at least one of our anti-activator regulons, only 31% of the QS-repressed genes were also repressed by deletion of at least one anti-activator (Tables 2, 4). Additionally, two genes of the QS-repressed regulon, PA3362 (encoding hypothetical protein AmiS) and PA4519 (encoding ornithine decarboxylase SpeC), were activated in all three anti-activator mutants compared with wild-type. The lack of regulatory overlap between the QS-repressed and anti-activator regulons suggests an additional, QS-independent pathway of regulatory activity for QteE and QslA.

**Figure 8:**
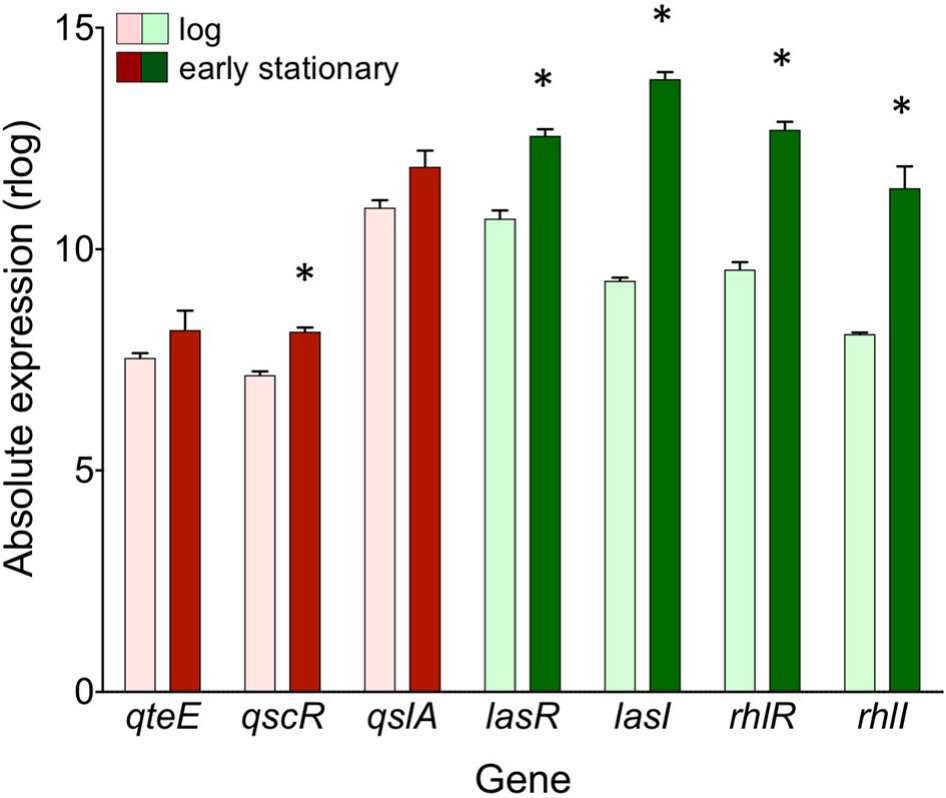
Absolute expression of genes coding for QS machinery and anti-activators in the wild-type. Absolute expression is presented as regularized log (rlog) values generated in DESeq2 using three biological replicates (*n* = 3). Absolute gene expression in log phase (light bars) and early stationary phase (dark bars) are grouped by gene as QS machinery (green bars) or anti-activators (red bars). Bars represent means + s.e.m. (*n* = 3). ^*^indicates significantly higher expression in early stationary phase than log phase, two-tailed *T*-test (α = 0.05).

Next, we specifically considered the expression of QS circuitry (*lasR, lasI, rhlR, rhlI*) and anti-activator (*qteE, qscR, qslA*) genes in log and early stationary phase in the wild-type. All R and I genes, including the anti-activator/orphan R gene *qscR*, showed significant increases in absolute expression in early stationary phase (α = 0.05, Figure 8) consistent with established mechanisms of QS autoregulation and stationary phase-dependent upregulation of R-genes (Schuster et al., 2003; Schuster and Greenberg, 2006). The expression of *qslA* and *qteE* was unchanged between log and early stationary phase. These expression patterns are consistent with the idea that the stoichiometry of R-proteins to anti-activators is key to QS target gene induction.

### Additional evidence linking quorum and anti-activator regulons

**Figure 9:**
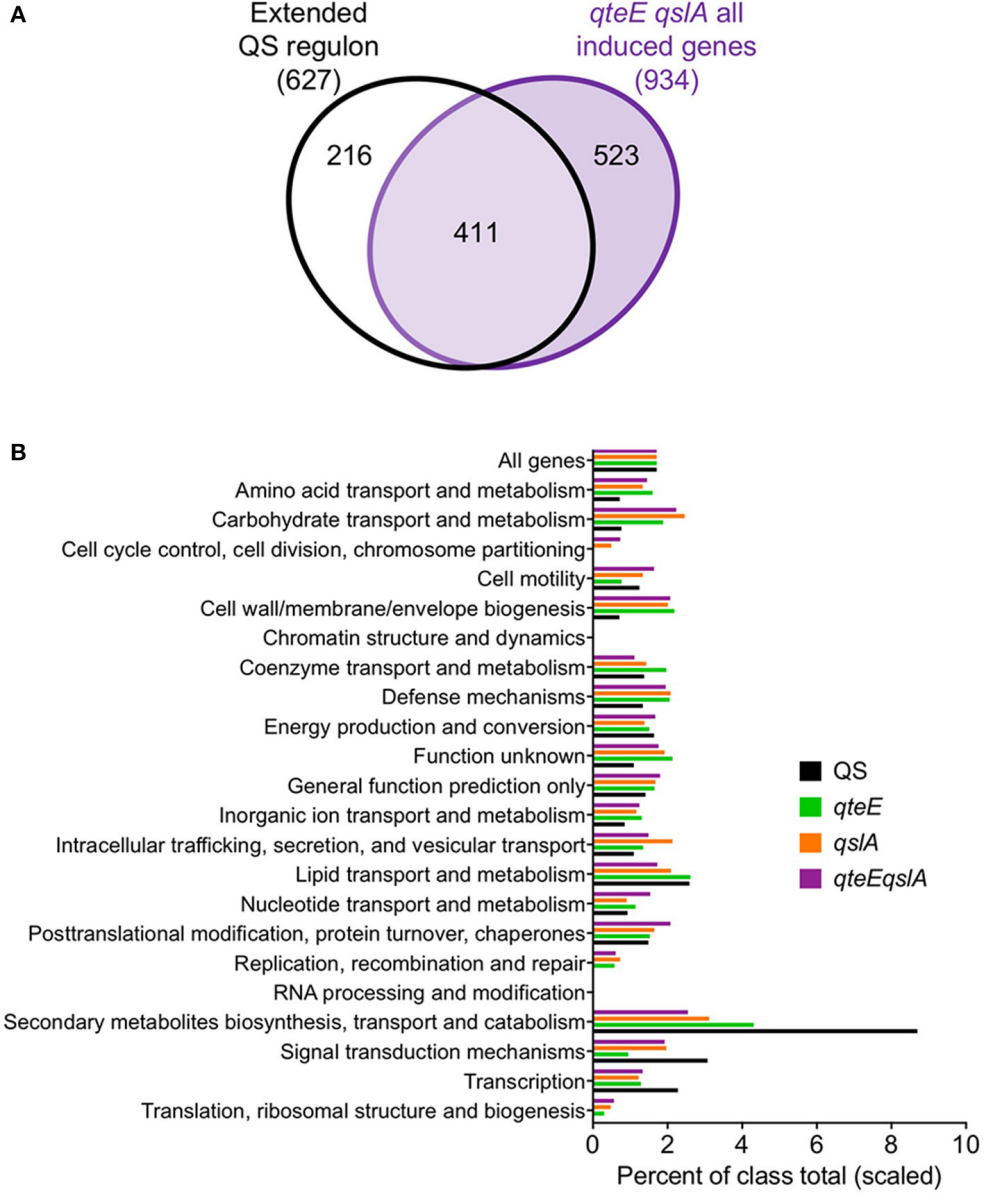
Broader context and functional relationship between quorum and anti-activator regulons. **(A)** Comparison of an extended QS regulon with genes induced in the *qteE qslA* anti-activator mutant in early stationary phase. The extended QS regulon contains all QS-induced genes identified in the current study and in previously published QS studies (Hentzer et al., 2003; Schuster et al., 2003; Wagner et al., 2003). Venn diagram is not scaled to gene number. **(B)** Functional classification of induced genes. Functional classes and annotations were retrieved from the Pseudomonas Genome Database. Bars represent percent of each functional class represented in induced gene lists, scaled to the size of the wild-type percentage of all genes (1.4%). Induced gene lists for each sample were assembled from differentially expressed (DE) genes in early stationary phase as determined in DESeq2 using three biological replicates (false discovery rate α = 0.05, *n* = 3).

The quorum-activated regulon determined in this study largely overlapped with genes affected by anti-activation, so we questioned whether other genes induced in the anti-activator mutants may be associated with other, previously identified QS gene sets (Hentzer et al., 2003; Schuster et al., 2003; Wagner et al., 2003). We assembled all genes identified as quorum-activated in this study and in the other three studies (Figure 5B). This yielded a list of 627 unique genes in an “extended” QS regulon. Comparison with the 934 genes induced in the *qteE qslA* mutant showed that 411 genes are shared with the extended QS regulon (Figure 9A). This overlap represents two-thirds of all genes of the extended QS regulon and nearly half of those induced in the *qteE qslA* mutant.

Lastly, we asked whether the large number of genes induced in the absence of anti-activators show a similar functional annotation profile to the WT QS regulon. We found generally good agreement in the functional distribution of genes between each anti-activator regulon (Figure 9B). Functional categories related to signal transduction, transcription, and secondary metabolite biosynthesis, transport and catabolism were more strongly represented in the WT QS regulon than anti-activator mutants. We also observed increases in typically underrepresented QS regulon categories in anti-activator mutants such as amino acid and carbohydrate transport and metabolism, as well as cell wall/membrane/envelope biogenesis. However, general observations show removal of anti-activators simply increased the number of genes in groups already represented in the WT QS regulon. This is consistent with the idea that most genes induced in the anti-activator regulons are QS-dependent, although more evidence is necessary to confirm this notion. It is plausible that anti-activator deletion allows an increase in the levels of active R-protein to an extent that is not normally achieved under physiological conditions. The set of genes activated under these conditions could then still be considered “quorum-sensing dependent”.

## Discussion

Anti-activation through binding of R-proteins is a mechanism that modulates the quorum-activation threshold in *P. aeruginosa* QS. It is part of a larger group of QS-dampening mechanisms that include transcriptional repression (RsaL) and dilution or environmental degradation of signal (De Kievit et al., 1999; Hense and Schuster, 2015). The currently known collection of anti-activator proteins, QteE, QscR, and QslA, was previously shown to have somewhat parallel effects in their roles of preventing premature activation of QS (Siehnel et al., 2010; Seet and Zhang, 2011; Gupta and Schuster, 2013; Chugani and Greenberg, 2014). Here, we demonstrate additive, overlapping effects for each anti-activator in the modulation of the quorum-activation threshold. Our results draw on evidence of QS promoter activity, QS phenotypes, and anti-activator transcriptional profiles. Our results paint a considerably more complex picture of the factors influencing the *P. aeruginosa* QS activation threshold than previously presented.

We found that deletion of anti-activator genes results in high-level activation of QS genes (Figures 2, 4, 7). This effect is a combination of increased expression rates and advanced expression at lower cell densities. Combination of certain deletions produced additive, if not synergistic effects on gene induction. The most dramatic rate increases and advanced expression were achieved by multi-deletion mutants lacking *qslA* (Figure 2). Deletion of *qslA* by itself produced the most modest increase in *lasB* expression among all single mutants, but additional deletion of either *qscR* or *qteE* in combination with *qslA* produced induction levels much higher than the combined deletion of *qscR* and *qteE* (Figure 2). These observations suggest a key role for QslA in determining the QS induction threshold, and a scenario where QslA may act via a molecular mechanism distinct from QscR or QteE. The expression patterns observed with the *lasB* reporter are mirrored at the transcriptome-level as anti-activator deletion advances the induction of most QS-activated genes from stationary to log phase (Figure 7, left panel). Taken together, our results indicate that some anti-activators have a stronger effect on the timing of QS induction than others. It is plausible that such combinatorial effects stem from protein-protein interactions between the anti-activators themselves and between anti-activators and R-proteins. *In vitro* binding studies involving all proteins in purified form would be most desirable for detailed mechanistic insights.

Our kinetic *P*_*lasB*_-*gfp* experiments showed expression skewed toward the earlier, presumably Las-controlled peak in all anti-activator mutants (including multiple deletions) with the exception of the *qslA* single mutant. Similar kinetic experiments conducted with both the wild-type and an isogenic *lasR* mutant showed the first expression peak disappears in the absence of LasR (Gupta and Schuster, 2013), providing support for the notion of sequential wild-type expression peaks corresponding to Las and Rhl system induction. In light of this evidence, our observations indicate that anti-activators may primarily target LasR rather than RhlR. Such a relationship is intuitive considering that induction of the Rhl system is generally subordinate to Las (Schuster and Greenberg, 2006). However, more direct evidence is needed to support this interaction model, as the independent effects of anti-activators on LasR and RhlR activity are not entirely clear. QteE is known to interact and destabilize both LasR and RhlR, but interaction with the latter was shown in the absence of LasR where competition between the two R-proteins for QteE binding was absent (Siehnel et al., 2010). QscR was also reported to associate with both LasR and RhlR *in vitro* in the absence of AHL using fluorescence anisotropy (Ledgham et al., 2003). However, direct evidence of the QscR-RhlR interaction *in vivo*, as well as the biological relevance of this association, is still needed. On the other hand, QslA was not shown to significantly abrogate RhlR-mediated transcription of *rhlI* in the *E. coli* heterologous host (Seet and Zhang, 2011), further supporting the Las-dominant interaction model described above.

Our transcriptome analysis produced a list of 79 quorum-activated genes, or roughly 1.4% of all *P. aeruginosa* genes, notably smaller than previous microarray studies that suggest “hundreds” of QS-activated genes (6–10% of genome) (Hentzer et al., 2003; Wagner et al., 2003; Schuster and Greenberg, 2006). Considering our choice of a semi-defined medium (CAA) that limits the final densities of bacteria to almost half that of previous studies (using LB broth), this difference is perhaps unsurprising. However, almost 90% of the genes we identified were also identified in at least one of the microarray studies (Figure 5B), supporting the notion of a core QS regulon conserved in *P. aeruginosa* suggested elsewhere (Schuster and Greenberg, 2006; Chugani et al., 2012). The large number of additional genes induced in anti-activator mutants could draw into question if these genes are all QS-dependent. It is possible that some of the genes identified as induced in anti-activator mutants are not regulated through canonical QS. Considering 56% of all genes induced in the strain lacking QteE and QslA were not shared with previously identified QS-activated gene sets (Figure 9A), a subset of these genes could conceivably be induced through a yet undetermined QS-independent mechanism. The very high secretion activity in anti-activator mutants would also require major changes in cellular metabolism that would indirectly affect gene expression on a massive scale.

It is equally possible that the large number of genes affected by simultaneous *qteE* and *qslA* inactivation, but not present in the extended QS regulon, are activated through canonical QS but are not induced in the native context as R-protein levels are not high enough. Such high levels of R-proteins may be achieved under certain physiologically relevant conditions, or they may be artificially high and lead to non-specific activation. In any case, the differences between each of our anti-activator regulons and the QS regulon could simply stem from the fact that each deletion results in a different level of free, active LasR: the higher the level of free LasR, the more promoters are bound and activated due to decreased competition for active LasR. This mechanism is most plausible with the nested differentially expressed genes identified in the log phase of growth in anti-activator mutants (Figures 4A, 6A), where almost all *qteE* genes are a subset of *qslA* genes, and almost all *qslA* genes are a subset of *qteE qslA* genes. Differential interaction of anti-activators with RhlR is equally plausible. Epistasis analysis could be used to address these possibilities, an approach that showed regulatory interactions in the functioning of parallel QS circuits in *V. harveyi* (Henke and Bassler, 2004). In our case, *lasR* and/or *rhlR* mutations would need to be introduced into strains harboring mutations in *qteE* and/or *qslA*. Such analyses could enable a better understanding of the regulatory interactions and dependencies of anti-activation in a QS-independent context. Transcriptome profiling experiments utilizing mutants lacking both anti-activators and LasR or RhlR, or both will allow exploration of this possibility. The relative contributions of LasR vs. RhlR could be further discerned by analyzing the QS-dependent effects of anti-activators on Las vs. Rhl-specific target genes.

LasR and RhlR QS receptors are generally understood to act as activators of transcriptional activity (Schuster et al., 2013), so we chose to focus most of our transcriptome analysis on genes induced in our DE analysis. However, our transcriptome analysis also found that 59 genes (1.1% of all *P. aeruginosa* genes) are repressed by QS in early stationary phase, similar to a previous microarray study of the *P. aeruginosa* QS regulon (Schuster et al., 2003). Quorum repression in general could be direct or indirect. For example, LasR or RhlR could activate downstream transcriptional repressors, or could repress some target genes directly, as has been shown for RhlR (Medina et al., 2003). The fraction of all DE genes repressed in anti-activator mutants approximately scaled with the sizes of the individual regulons, similar to genes that were induced (Table 2). However, this set of genes was largely independent of the genes repressed by QS, indicating anti-activator effects that are independent of QS. It is possible that anti-activator proteins interact with regulatory proteins other than LasR and RhlR, or that they function in entirely different ways. The additional activity of QscR as a transcriptional activator has already been noted (Lee et al., 2006).

Evaluation of functional annotations of induced genes in our strains showed few substantive differences in their overall functional class distribution, and all were largely similar to the quorum-activated regulon distribution (Figure 9B). Our results were generally consistent with previous analyses of the content of QS regulons, with the exception of a previously reported category of secreted factors (Schuster et al., 2003; Schuster and Greenberg, 2006). However, a revision of the functional categories at pseudomonas.com since the publication of those reports in part explains this discrepancy. QS is responsible for global gene regulation in *P. aeruginosa* (Schuster and Greenberg, 2006), including genes involved in growth and central metabolism, biosynthesis and transport of secondary metabolites, and signal transduction mechanisms. So, our findings are generally in support of the proposed mechanisms of anti-activators as suppressors of QS regulon expression.

Cooperative secretions provide a collective, density-dependent benefit but are costly to produce for the individual. As cooperative secretions are common in QS regulons (Hense and Schuster, 2015), the precise tuning of the induction threshold is therefore critical for population fitness (Pai and You, 2009; Pai et al., 2012). Lack of anti-activation may waste resources through overinvestment in secretion, and cause exploitation of these secretions by neighboring cells. In *P. aeruginosa*, increased expression of LasB elastase through individual deletion of *qteE* or *qscR* enhances growth in media requiring QS-controlled proteolysis, but also imposes a fitness cost when QS is not required, and increases exploitation by non-producing cells (Gupta and Schuster, 2013).

The presence of multiple anti-activators may help tune the QS induction threshold in response to the physical and social environment. Thus, we might expect differential regulation of anti-activator expression in different contexts. In our study, we have merely explored one growth medium and two growth phases, revealing generally constitutive expression (Figure 8). In a separate study, qRT-PCR analysis of *qslA* transcription also showed constitutive expression in LB medium (Seet and Zhang, 2011). The regulatory dynamics of *qteE* and *qscR*, in addition to those of *qslA* under varying growth conditions, are not clear. QscR is different from other anti-activators in that it can also respond to AHL signals and effectively act as a transcriptional activator on its own (Lequette et al., 2006). QscR exhibits promiscuity in its response to AHL signals; in addition to 3OC12-HSL generated by LasI, QscR responds to AHLs produced by other bacteria, adding an additional layer of complexity to QscR activity (Lee et al., 2006). Transcription of *qscR* may also be under tighter control than other anti-activators. For example, *qscR* transcription is regulated by both the global regulator VqsR (Liang et al., 2012) and LasR itself.

Sequestration of R-proteins by anti-activation may be key in preventing signal short-circuiting within cells, as suggested by mathematical modeling (Goryachev et al., 2005). Maintaining true QS and preventing costly, constitutive expression of QS-controlled products may warrant multiple “failsafe” anti-activation mechanisms. However, our results indicate that the three anti-activators do not merely function redundantly; rather we found a range of combinatorial effects when more than one anti-activator is deleted. In addition, removal of even all three anti-activators does not lead to short-circuiting under our culture conditions. Additional mechanisms that prevent short-circuiting would be those that keep intracellular AHL levels sufficiently low. These include *lasI* feedback inhibition by RsaL (Rampioni et al., 2007; Bondi et al., 2017) and active efflux of 3OC12-HSL via MexAB-OprM (Pearson et al., 1999). It is also plausible that other anti-activators remain to be identified, or that other culture conditions may trigger a short-circuiting effect (Van Delden et al., 2001).

We conclude that the anti-activators QteE, QscR, and QslA differentially reduce the magnitude and delay the timing of QS-gene activation and subsequent virulence factor production. This effect is dependent on the specific combination of anti-activators present, with QslA in combination with another anti-activator conferring the greatest effect. Anti-activators affect an overlapping but distinct set of genes largely governed by QS, and do so in a combinatorial fashion. This study further supports the concept of a core QS regulon in *P. aeruginosa*, and provides the ground work for multiple directions of fundamental investigation of anti-activation and gene regulation in bacteria. Our transcriptome results will likely aid studies seeking to determine the roles of anti-activators in *P. aeruginosa* pathogenesis, clinical avenues for inhibiting QS, and regulation of virulence gene expression. More broadly, our results will also contribute to a more detailed understanding of the factors influencing the QS threshold in diverse bacteria.

## Author contributions

The author(s) have made the following declarations about their contributions. Conceived and designed the experiments: KA and MS. Performed the experiments: KA. Analyzed the data: KA. Contributed reagents, materials, analysis tools: KA and MS. Wrote the paper: KA and MS.

### Conflict of interest statement

The authors declare that the research was conducted in the absence of any commercial or financial relationships that could be construed as a potential conflict of interest.

## Supplementary material

Table S1 All differentially expressed genes in Log and Early stationary phase comparisons.
